# Integrating Conservation and Community Engagement in Free-Roaming Cat Management: A Case Study from a Natura 2000 Protected Area

**DOI:** 10.3390/ani15030429

**Published:** 2025-02-04

**Authors:** Octavio P. Luzardo, Andrea Hansen, Beatriz Martín-Cruz, Ana Macías-Montes, María del Mar Travieso-Aja

**Affiliations:** 1 Research Institute of Biomedical and Health Sciences (IUIBS), University of Las Palmas de Gran Canaria, Paseo Blas Cabrera “Físico” s/n, 35016 Las Palmas de Gran Canaria, Spain; beatriz.martin@ulpgc.es (B.M.-C.); ana.macias@ulpgc.es (A.M.-M.); marimar.travieso@ulpgc.es (M.d.M.T.-A.); 2 Spanish Biomedical Research Center in Physiopathology of Obesity and Nutrition (CIBERObn), 28029 Madrid, Spain; 3 Association for Respect and Commitment to Animals and Nature (ARYCAN), 35217 Valsequillo de Gran Canaria, Spain; arycan.ah@gmail.com

**Keywords:** human–wildlife conflict, non-lethal management, protected areas, trap–neuter–return, TNR, feral cats, invasive species, conservation policy

## Abstract

La Graciosa, a Natura 2000 site in the Canary Islands, faces substantial conservation challenges, including a large free-roaming cat population that threatens the island’s native biodiversity. In July 2024, a Trap–Neuter–Return (TNR) campaign achieved an 81.4% sterilization rate within urban areas, highlighting TNR’s short-term effectiveness in reducing reproductive potential and, consequently, mitigating predation pressures primarily through the prevention of new litters and reduced reproductive activity in cats. The campaign’s success relied heavily on the active involvement of the local community, who assisted with identifying, trapping, and monitoring free-roaming cats, thereby facilitating a high sterilization rate. However, administrative restrictions hindered access to peri-urban zones, leaving essential population clusters unsterilized and limiting the campaign’s overall scope. Additionally, strong opposition from conservation groups, amplified by extensive media coverage, halted the project prematurely, reducing the effective sterilization rate to 69.3% within three months. Population Viability Analysis (PVA) suggests that achieving high sterilization rates could lead to population reduction over time; however, the inability to access all population segments and to reach the ideal 93–95% sterilization threshold limits TNR’s potential as a long-term standalone solution. Our findings underscore the need for adaptive, context-specific management frameworks in ecologically sensitive areas that integrate TNR with complementary measures, consider regulatory barriers, and value community involvement. This case study provides crucial insights for policymakers and conservationists seeking to balance biodiversity conservation with humane management practices in protected areas.

## Simple Summary

This study evaluates the effectiveness of a Trap–Neuter–Return (TNR) program implemented in La Graciosa, a Natura 2000 protected area in the Canary Islands, to manage its free-roaming cat population. The campaign achieved an 81.4% sterilization rate in urban areas within three days, significantly reducing the cats’ reproductive potential and contributing to an alleviation of predation pressures on vulnerable species. Active community involvement played a critical role in the campaign’s success, yet administrative restrictions and opposition from conservation groups limited its scope. A Population Viability Analysis (PVA) revealed that while high sterilization rates can reduce populations, achieving the necessary 93–95% threshold is essential for long-term success. The study underscores the need for adaptive, context-specific management strategies that integrate TNR with complementary measures like adoption or relocation and highlights the challenges posed by regulatory and societal factors in balancing biodiversity conservation with humane management practices.

## 1. Introduction

The domestic cat (*Felis catus*), despite its domestication and millennia-long coexistence with humans, is recognized as one of the most ecologically impactful introduced species, significantly affecting biodiversity in non-native environments [1,2,3]. On a global scale, cats have established populations on all continents except Antarctica and on hundreds of islands, where they pose a major threat to native biodiversity, particularly within sensitive and isolated ecosystems [1,4]. Numerous studies have linked free-roaming cats to the decline and extinction of various species, from ground-nesting birds to small reptiles and mammals, leading to their recognition as one of the world’s 100 species with the most significant ecological impact [5,6].

Although biodiversity loss is driven by multiple factors, such as habitat destruction, climate change, and the introduction of other non-native predators [7,8,9,10], the presence of free-roaming cats has emerged as a prominent driver, especially in insular environments where endemic species are particularly vulnerable [1,11]. Beyond direct predation, their impacts extend to subtle ecological shifts, including trophic cascades and hyperpredation, whereby the presence of introduced prey such as rodents enables higher cat densities, which in turn amplify predation pressure on native species [12]. Therefore, managing introduced species like free-roaming cats poses unique challenges, especially in ecosystems with high ecological sensitivity and complexity. For this reason, the presence of free-roaming cats in protected areas such as Natura 2000 sites is fundamentally incompatible with the conservation of biodiversity, particularly in ecologically fragile environments. Managing these populations is essential to minimize their impact while ensuring the long-term preservation of native ecosystems.

In response, recent studies emphasize ecosystem-level approaches to mitigate unintended consequences. For instance, models of invasive predator control, such as those applied on Phillip Island, Australia, suggest that controlling prey species (e.g., rodents) rather than top predators can yield more sustainable outcomes by accounting for complex interspecies interactions [13]. The dual threat posed by free-roaming cats—relying on anthropogenic resources while preying on native fauna—underscores the need for conservation strategies that balance ecological goals with human dimensions. These human aspects include the emotional bonds, sense of responsibility, and roles that local communities attribute to these animals [14,15,16,17,18,19].

La Graciosa—our study focus—serves as a fitting example of this complex challenge. This island, part of the Natura 2000 network, hosts free-roaming cat populations concentrated around human settlements [20], which rely heavily on anthropogenic resources while intersecting with vulnerable ecosystems. Situated north of Lanzarote in the Canary Islands, La Graciosa is the largest and only inhabited island of the Archipiélago Chinijo, a natural area that also includes Montaña Clara, Roque del Este, Roque del Oeste, and Alegranza. These islets and the Famara Massif were designated a Natural Park in 1987 [21] and subsequently included in the Natura 2000 Network as a Site of Community Importance (SCI), later achieving status as a Special Area of Conservation (SAC) and Special Protection Area (SPA) for birds [22,23].

However, the island faces substantial anthropogenic pressures, including an annual influx of approximately 500,000 tourists facilitated by frequent ferry services, with no restrictions on visitor numbers or accompanying pets. Additionally, more than 400 vehicles—many used for informal tourism purposes—traverse the island’s delicate terrain, further straining its ecosystems. The situation is further compounded by invasive species, including *Rattus rattus* (black rats), *Mus musculus* (house mice), *Oryctolagus cuniculus* (European rabbits), *Canis familiaris* (hunting and pet dogs), among others, which disrupt local habitats. The long-standing presence of cats since the mid-1800s further compounds these ecological challenges. Thus, the cumulative impact of these pressures highlights the urgent need for an adaptive management framework to preserve the island’s ecological integrity [24]. These cumulative impacts highlight the urgent need to manage free-roaming cats as part of a broader conservation strategy to restore the ecological integrity of the island, while prioritizing non-lethal and scientifically sound methods that align with legal frameworks.

Efforts over the past two decades to manage the free-roaming cat population on La Graciosa, within the existing biodiversity conservation framework [25,26,27], have encountered numerous obstacles, primarily due to fragmented governance across multiple administrative entities. The Government of Spain, the Government of the Canary Islands, the Cabildo of Lanzarote, the municipality of Teguise, and the Canary Islands Ports Authority each have oversight of different aspects of the island, with limited inter-agency coordination [24].

Additionally, the local community has shown resistance to external management interventions, perceiving cats as essential for controlling rodent and cockroach populations. Despite community calls for authorities to sterilize free-roaming cats—many of which are fed by local caretakers—certain administrations opposed such measures, citing that trapped cats would either be culled or placed in holding facilities. This stance has conflicted with local opposition to lethal measures, a lack of permanent housing facilities for cats, and the saturation of animal welfare organizations. Consequently, administrative inaction has become the dominant approach in urban areas, enabling unchecked reproduction without an official census or management plan. By contrast, only a few dozen cats have been culled in protected areas over the last two decades, yielding negligible impacts on population control.

The recent enactment of Spain’s Law 7/2023 on animal welfare marks a pivotal shift in the legal landscape of cat population management. This law mandates that all municipalities implement management plans, redefining “community cats” to include all outdoor cats, regardless of socialization or habitat, thereby removing their classification as invasive alien species [28]. Law 7/2023 endorses the Trap–Neuter–Return (TNR) method and the adoption of sociable cats as the primary approach for managing cat populations in urban and peri-urban areas, as well as in natural areas, where relocation of certain cats is permitted under well-documented conditions. Additionally, the law explicitly prohibits lethal management practices, such as culling, and the indefinite confinement of cats, limiting the options available for controlling populations in natural spaces. These provisions aim to prioritize humane treatment, but they also pose challenges in addressing the ecological impacts of community cats within sensitive ecosystems. Hereafter, we will refer to free-roaming cats as “community cats”, in alignment with the legal definition provided by Law 7/2023.

Nonetheless, the application of Law 7/2023 in natural areas, particularly within the Natura 2000 network, remains controversial due to differing interpretations. While some advocates argue that Law 7/2023 should apply broadly, others assert that protected spaces should prioritize ecological integrity, highlighting a potential regulatory gap. This nuanced approach contrasts with the provisions of Law 42/2007, which supports removing non-native species to preserve biodiversity [25,26]. The coexistence of these laws, combined with historical inaction and community resistance to lethal management, presents significant challenges for achieving coherent and effective conservation strategies. This duality underscores the critical need for integrated policies that reconcile animal welfare considerations with biodiversity conservation priorities.

Protected natural areas, like La Graciosa, offer valuable testing grounds for conservation models with broader applications. Specifically, La Graciosa provides an opportunity to assess the efficacy of TNR as mandated by Law 7/2023, given the limited influence of immigrant or abandoned cats. However, due to ongoing controversy over the law’s application in natural spaces, this study concentrated on the island’s urban areas (also part of the Natura 2000 protected area) where we obtained legal permission. The objectives of our study were to (i) conduct a comprehensive census of the cat populations in the human settlements of Caleta de Sebo and Pedro Barba; (ii) implement a short-term, intensive mass sterilization campaign; (iii) continue sterilizations over subsequent months to approach a 100% sterilization rate; and (iv) monitor population changes to evaluate the impact of TNR.

## 2. Materials and Methods

### 2.1. Study Area

**Figure 1:**
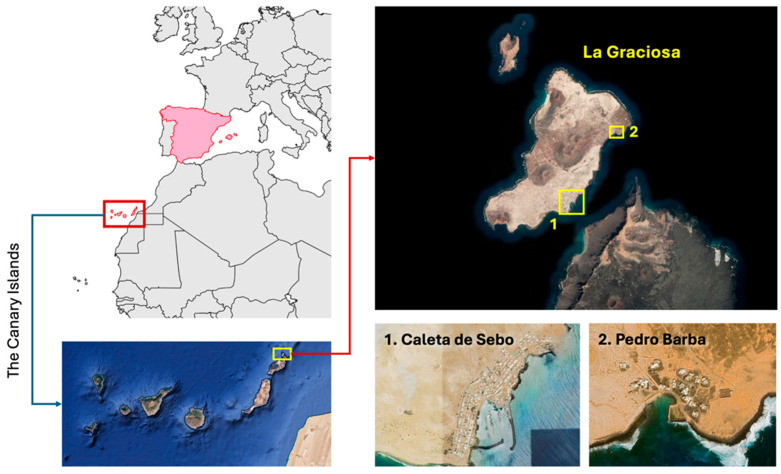
The location of La Graciosa within the Canary Islands (red square in the upper left panel) and its primary settlements. La Graciosa (yellow square in the lower left panel), part of the Natura 2000 network, is situated north of Lanzarote in the Canary Islands. The island’s main settlements, Caleta de Sebo (1) and Pedro Barba (2), are highlighted (yellow squares in the upper right panel). Caleta de Sebo serves as the primary residential and administrative center, while Pedro Barba functions as a smaller residential area. Both human settlements also belong to the Natura 2000 protected area.

This study was conducted exclusively within the urban settlements of La Graciosa, located in the Canary Islands, Spain (29.2500° N, 13.5000° W) (Figure 1). La Graciosa is characterized by an arid landscape dominated by volcanic formations, including extinct cones and lava fields. The island has a total area of 29 km^2^, making it the smallest inhabited island of the Canary Archipelago. Its coastline varies from sandy beaches in the south to rugged rocky shores in other areas, creating diverse habitats that support unique ecological communities. La Graciosa is part of the Chinijo Archipelago Natural Park, which includes several islets and covers a total protected area of approximately 700 km^2^, mostly marine ecosystems.

The primary urban settlement is Caleta de Sebo, the main population center and administrative capital, housing 720 permanent residents in 2023 [29]. The village features traditional low-rise buildings and unpaved sandy streets. Infrastructure is limited; notably, there is no sewage system or wastewater treatment plant, which poses challenges for environmental management. Caleta de Sebo serves as the principal entry point for tourists arriving via regular ferry services from Lanzarote. The high influx of visitors—recorded at nearly 600,000 passengers annually in 2023 [30]—exerts significant anthropogenic pressure on local ecosystems. The tourism sector heavily influences the local economy, evidenced by a high ratio of vacation rentals to residents [31]. The secondary settlement is Pedro Barba, situated on the northeastern coast. Historically the first inhabited area of the island, it currently has only two permanent residents, with most structures functioning as temporary accommodations for tourists.

These urban areas are surrounded by ecologically significant zones that are part of the Natura 2000 Network, designated as both a Special Area of Conservation (SAC) and a Special Protection Area (SPA) for birds [23].

### 2.2. Legal Permits and Ethical Approvals

The implementation of this study required securing various legal permits and ethical clearances in accordance with national and regional regulations. The initial authorization was obtained from the Environmental Council of the Cabildo of Lanzarote, allowing a census and ecological study of community cats across La Graciosa (Resolution 2868/2024). Due to the concentration of cats in the urban areas of Caleta de Sebo and Pedro Barba, as well as anticipated administrative challenges, the study’s focus was shifted to these locations under the jurisdiction of Teguise Municipality. This approach aligns with the provisions of Law 7/2023 on animal welfare, which assigns community cat management responsibilities to local municipalities [28,32]. Thus, permits were also obtained from the Teguise Municipality (reference 2024-005133) and the Canary Islands Ports Authority (reference 1168/2024), due to the presence of cats within port facilities, to ensure compliance with their management protocols. The College of Veterinarians of Las Palmas granted authorization for setting up a temporary veterinary hospital, a necessity given the absence of permanent veterinary infrastructure on the island. Ethical approval for the collection of biological samples during the surgical procedures was granted by the Ethics Committee for Animal Experimentation at the University of Las Palmas de Gran Canaria (Resolution OEBA_ULPGC_35/2023).

### 2.3. Pre-Sterilization Census

An experienced field researcher was stationed on La Graciosa from 12 February to 15 May 2024, to carry out a comprehensive pre-sterilization census of the cat population in preparation for the sterilization campaign. The researcher’s extended presence on the island was crucial for building trust within the local community, which initially displayed resistance due to past management practices that had eroded confidence. Through semi-structured, informal door-to-door interviews, the researcher established rapport with local cat owners and caretakers, facilitating the collection of essential information regarding feeding sites, schedules, and individual cats.

The researcher accompanied caretakers during feeding times to observe and confirm the presence of specific cats, accounting for their movement between different feeding points, particularly in Caleta de Sebo. Each cat was photographed to ensure accurate identification. Basic sociodemographic data of caregivers and owners were also gathered, including age range, gender, and their relationship to the cats (owner, caretaker, or occasional feeder). Information on the number of cats in their care and their willingness to participate in the sterilization program was collected as well.

For each cat, detailed data were recorded, including physical characteristics such as sex, estimated age, coat color and pattern, fur length, and breed (if identifiable). The reproductive status (neutered or unneutered) was noted, along with the owner’s or caretaker’s interest in sterilization and willingness to cover associated costs. Behavioral aspects were documented, including the cat’s access to the outdoors (indoor, indoor/outdoor, outdoor), whether it was kept indoors at night, and its origin (born in house, found, gifted, adopted, or purchased). The purpose of keeping the cat—such as companionship, pest control, or compassion—was also recorded. Feeding practices were detailed, noting the type of food provided (commercial cat food, kitchen leftovers, homemade meals), feeding frequency, and feeding locations. Health care information included the frequency of veterinary visits and the use of antiparasitic treatments. Additionally, the presence of a collar or other forms of identification was recorded to distinguish owned cats from unowned ones.

Community cats that frequented public areas such as restaurants, food outlets, and waste disposal sites were also registered and included in the census.

### 2.4. Mass Sterilization Campaign

The mass sterilization campaign was conducted over eight days from 22 July to 29 July 2024, aiming to significantly reduce the community cat population on La Graciosa through a coordinated Trap–Neuter–Return (TNR) approach. Meticulous logistical planning was undertaken, involving the transport of specialized equipment and assembling a team of 18 professionals, including veterinarians, veterinary technicians and researchers from the University of Las Palmas de Gran Canaria, and experienced trapping volunteers.

**Figure 2:**
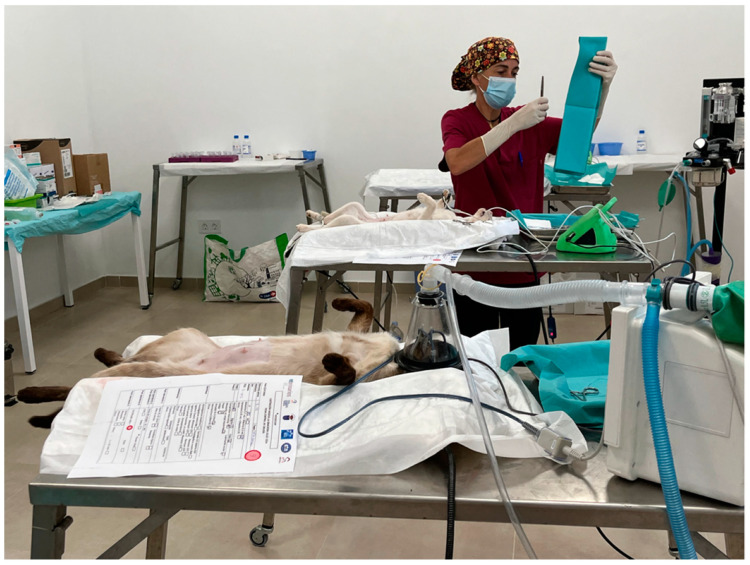
Surgical area setup during the mass sterilization campaign. The room was equipped with three operating tables, anesthesia machines, surgical lights, and patient monitoring equipment, ensuring optimal conditions for high-quality, high-volume surgical procedures.

A temporary veterinary facility was established at the sociocultural center in Caleta de Sebo, organized into areas for reception, pre-surgical examination, surgery, and post-operative care. To implement a high-volume, high-quality spay–neuter approach, the surgical area was specifically organized and equipped with essential veterinary surgical equipment to ensure high standards of animal care [33] (Figure 2).

During the campaign, each cat was photographed from multiple angles—frontal, left lateral, right lateral, and headshots—to ensure accurate identification. Cats were also marked with a left ear tip, following standard TNR methodology, and microchipped. Post-surgical management included vaccination, deworming, microchipping, and monitoring until full recovery. The biological samples collected included blood, swabs, fecal samples from cats that defecated during the process, hair and whisker (vibrissae) samples, and the ear tissue removed during the left ear-tipping for identification marking. These samples were gathered for future analyses related to the efficacy of the TNR program. After recovery, cats were returned to their original locations, with GPS coordinates recorded to ensure accurate release sites [34,35].

Trapping was methodically planned and executed in collaboration with local cat caretakers, who played a pivotal role in identifying trapping sites and schedules. Humane live traps (Tomahawk Live Trap Co., Ltd., Hazelhurst, WI, USA) were used to minimize stress and injury to the cats [34,35]. Prior to trapping, a public information campaign was implemented, including distributing flyers to advise residents and visitors to refrain from feeding the cats starting the evening of 24 July to increase trapping success.

Surgical sterilizations were performed over three consecutive days (26–28 July) following standardized veterinary protocols in accordance with international best practices. Procedures included general anesthesia, aseptic techniques, and pain management protocols to minimize stress and promote recovery. The cats were closely monitored post-surgery and only released once deemed fit [33].

### 2.5. Post-Sterilization Monitoring

Following the mass sterilization campaign, plans were established to continue sterilizations of the remaining unsterilized cats, particularly those difficult to capture during the initial effort. A complete surgical station was left on the island, and arrangements were made with municipal authorities to provide a temporary facility to function as a veterinary clinic. This setup was intended to facilitate the ongoing trapping and sterilization of cats without dedicated caregivers, which primarily fed on restaurant leftovers or waste from garbage bins. However, external factors, including public and administrative challenges, hindered the project’s continuity, preventing the completion of the planned sterilizations.

Despite these challenges, monitoring of the cat population continued through a trained volunteer who remained on La Graciosa from 16 August to 6 November 2024. The volunteer was responsible for tracking the status of the sterilized cats and identifying the unsterilized individuals. Close collaboration with the local resident community was maintained to update the census, carefully recording new births, pregnancies, deaths, and adoptions.

A new data sheet was developed to record specific information for the unsterilized cats, and extensive photographic records were collected to aid in future identification and potential capture efforts. An estimated count of cats in peri-urban areas, including the waste disposal site and agricultural plots within the island’s natural reserve, was conducted despite restrictions on direct intervention due to administrative boundaries beyond municipal jurisdiction. Documenting these populations was essential to understand the broader dynamics of cat movement and density across the island.

Attempts were made to habituate these cats to human presence by offering attractive treats to facilitate future trapping; however, further captures were not realized due to external constraints.

### 2.6. Population Dynamics Simulation Model

To predict the long-term outcomes of the sterilization campaign and future population trends of community cats on La Graciosa, we conducted a Population Viability Analysis (PVA) using the individual-based stochastic simulation software VORTEX version 10.6.0 [36]. This tool allowed us to model the demographic dynamics of the cat population under various management scenarios, incorporating local conditions and specific interventions.

Estimating the carrying capacity was essential, given that the current community cat populations in La Graciosa’s urban centers, particularly Caleta de Sebo, are known to exceed sustainable thresholds. Evidence of cats venturing into surrounding natural environments indicates that the urban areas alone cannot support the current population levels, which has subsequent impacts on adjacent ecosystems. In our model, we set the carrying capacity at 2.5 times the initial censused population in each settlement, representing a worst-case scenario. This value reflects both the growing tourism industry and ongoing urban development on the island, which contribute to an increasing availability of resources for community cats. Over the past year, new restaurants have opened, additional tourist accommodations have been built, and there is consideration for expanding ferry services, all of which are expected to increase anthropogenic food sources. Moreover, plans for the installation of a sewage system are likely to indirectly promote the growth of rodent populations, providing additional prey for cats. While these factors may not lead to an immediate increase in cat populations, they justify using a conservative carrying capacity estimate of approximately 460 cats island-wide, aligning with the island’s 29 km^2^ area and potential resource expansion.

The model considered two subpopulations within each settlement: sterilized and unsterilized cats. Initial demographic parameters, including age and sex distributions, were derived from the pre-sterilization census. For Pedro Barba, while in one of the scenarios only one adult unneutered female cat was recorded (post TNR intervention), we assumed potential immigration from Caleta de Sebo, approximately 3.5 km away, given the accessible trails and observed movement of male cats during mating seasons. This assumption aligns with ecological observations of inter-settlement dispersal among community cat populations. Reproductive and mortality rates, along with disease prevalence, were informed by empirical data from over 15 years of feline management in the Canary Islands and relevant literature [37,38,39,40]. The PVA assumed continuous breeding throughout the year, reflecting the stable climate of the Canary Islands. Sterilization was modeled as a catastrophe in reproductive output for the sterilized subpopulation. Potential disease outbreaks were included as stochastic events affecting mortality for both subpopulations in each location, adding realism to the model by accounting for health-related fluctuations. All these data are summarized in Supplementary Table S1.

Over a 10-year period, we simulated three scenarios, each repeated for 1000 iterations to ensure robust projections. The first scenario represented a baseline without any management interventions, allowing the cat population to grow unchecked and naturally. The second scenario modeled the outcomes following the intervention, incorporating the sterilization rate achieved during the campaign in urban areas. Lastly, the third scenario projected the effects of sustained management efforts aimed at reaching and maintaining an ideal 93–95% sterilization rate, reflecting continuous follow-up actions. These simulations provided comparative insights into population trends under different management strategies.

## 3. Results

### 3.1. Population Census and Structure

The pre-sterilization census conducted from February to May 2024 provided detailed data on the community cat populations in Caleta de Sebo and Pedro Barba. In Caleta de Sebo, a total of 152 unowned and 24 owned cats were recorded. The gender distribution included 73 females, 65 males, and 46 individuals whose sex was undetermined due to their young age or limited proximity for observation. Most cats were estimated to be between 1 and 5 years old. Within the unowned population, 28 cats were neutered, while 124 remained unneutered. The owned population comprised 9 neutered and 15 unneutered cats (Table 1). The census included at least 35 kittens, although this number was likely underestimated as some may have been hidden by their mothers. This assumption was supported by the presence of two lactating females observed during the final days of the census. Additionally, at least 3 females were visibly pregnant, indicating ongoing reproduction within the unowned population. All unowned cats were outdoor-only (community cats), while among the owned cats, 8 were kept exclusively indoors, and 16 had both indoor and outdoor access (also considered as community cats, according to the new legal framework in Spain). Most cat owners and caretakers supported a Trap–Neuter–Return (TNR) strategy; however, the majority were not willing to cover the costs of the surgical intervention, with only two individuals preferring no intervention at all.

In Pedro Barba, the census documented a smaller group of 8 unowned cats, consisting of 5 males and 3 females. All cats in this settlement were outdoor-only, highlighting the simpler and more homogeneous nature of this population (Table 1).

**Figure 3:**
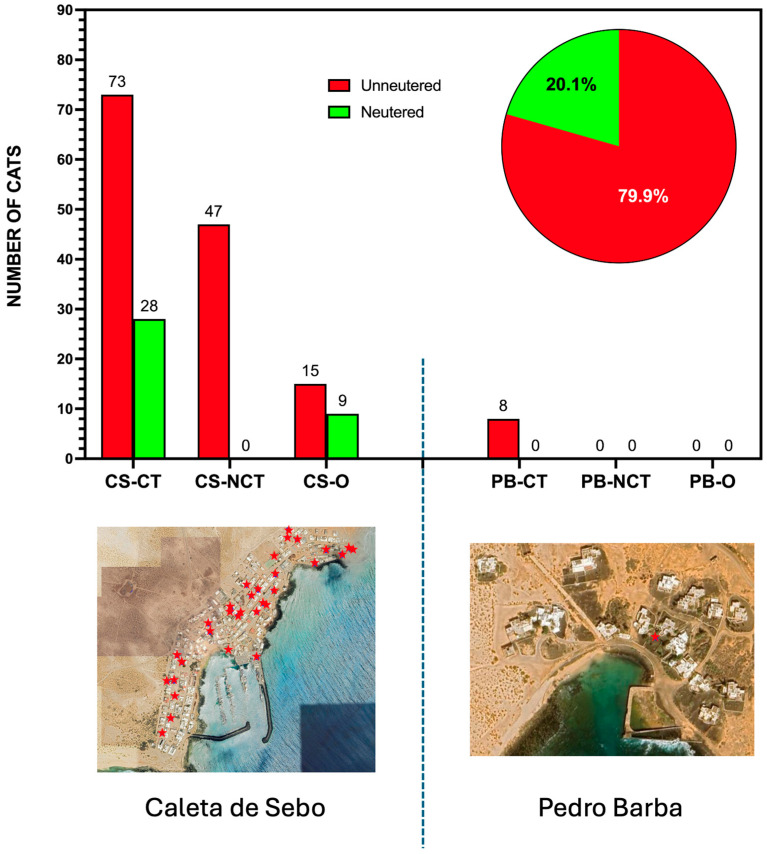
The distribution of neutered and unneutered cats across different categories in Caleta de Sebo (CS) and Pedro Barba (PB) on La Graciosa. The bar chart represents the number of neutered (green) and unneutered (red) cats within community-tended (CT), non-community-tended (NCT), and owned (O) groups in both settlements. The pie chart shows the overall sterilization status of the community cat population, with 20.1% neutered and 79.9% unneutered. The maps of Caleta de Sebo and Pedro Barba indicate the feeding points (red stars) and the spatial distribution of community cats in urban areas, where the intervention was focused.

In Caleta de Sebo, the proportion of unneutered cats was notably higher than that of neutered ones, as depicted in Figure 3. Among the unowned cats, most fell into the community-tended groups (CS-CT), where 73 individuals were unneutered compared to 28 that were sterilized. The non-community-tended group (CS-NCT) included 47 unneutered cats with no sterilized individuals recorded. Owned cats (CS-O) displayed a more balanced distribution, with 15 unneutered and 9 neutered cats. In Pedro Barba, the entire identified population of 8 unowned cats was unneutered, emphasizing the lack of intervention in this smaller settlement. The overall data depicted in Figure 3 clearly demonstrate that unneutered cats represented the majority across both urban areas, highlighting the need for targeted management strategies.

We assessed the management scenarios through an individual-based, stochastic simulation model to project the long-term viability of the community cat populations in Caleta de Sebo and Pedro Barba. The PVA conducted with Vortex 10.6.0 revealed distinct outcomes for each settlement.

**Figure 4:**
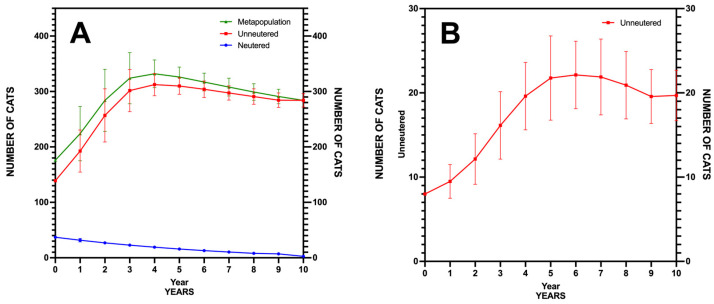
Projected population dynamics for community cats in Caleta de Sebo (**A**) and Pedro Barba (**B**) on La Graciosa based on Population Viability Analysis (PVA) simulations over 10-year period.

In Caleta de Sebo, the simulation based on 1000 iterations showed a strong growth rate (r = 0.382), indicative of exponential population increase if current conditions persist. The variability in growth rate (SD(r) = 0.199) indicated moderate annual fluctuations, suggesting that environmental or demographic randomness could affect population numbers. The model predicted no likelihood of eradication (Pr. Extinction = 0.00) over the 10-year period, with the mean population size projected to reach 284 individuals, emphasizing substantial growth from the initial figures (Figure 4A).

For Pedro Barba, the results demonstrated a slower yet positive growth rate (r = 0.317), pointing to gradual population expansion. The standard deviation of the growth rate (SD(r) = 0.238) suggested considerable variability, signaling potential year-to-year changes influenced by stochastic factors. The probability of extinction was noted at 0.04, translating to a 4% risk of local extinction over the decade, highlighting the vulnerability associated with smaller populations. The projected mean population size (N = 21) reflected a steady increase from the initial eight individuals, showing growth, albeit with more caution compared to Caleta de Sebo (Figure 4B).

### 3.2. Outcomes of Sterilization Campaign

The sterilization campaign conducted over three days in July 2024 involved 18 volunteers, local cat caretakers, and significant participation from the local community in La Graciosa. The campaign resulted in the intervention of 126 cats, with 113 undergoing sterilizations, including 99 unowned and 14 owned (Table 2). Additionally, 5 kittens from lactating females that were trapped during the campaign were placed for adoption, and the sterilization of 10 visibly pregnant females prevented the birth of approximately 39 kittens.

Of the 126 cats intervened, 13 were already sterilized prior to the campaign (7 unowned and 6 owned). These individuals did not require additional sterilization but received microchip implantation, registration, sanitation, vaccination, and deworming. The sex distribution among these previously sterilized cats included 4 males and 3 females among the unowned cats and 5 males and 1 female among the owned cats.

Over the three days, trapping results were notable: 54 cats were captured on the first day, followed by 42 on the second, and 27 on the final day. While most captures relied on trapping, on the second and third days, some cat owners cooperated by having their cats ready for the team in case the traps did not yield enough captures. The total gender distribution comprised 54 males and 59 females, with 10 of the females visibly pregnant. Most cats were classified with a body condition score of 5–6, indicating a generally adequate physical condition.

**Figure 5:**
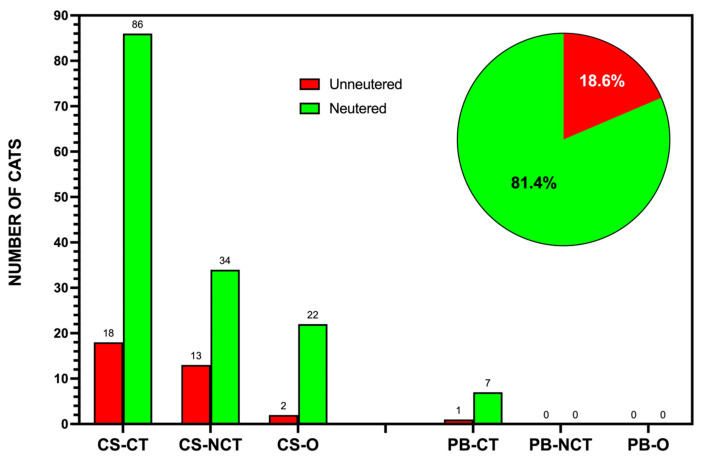
The effectiveness of the July 2024 sterilization campaign in La Graciosa, Canary Islands. The bar chart shows the number of neutered (green) versus unneutered (red) cats recorded across different feeding points in Caleta de Sebo and Pedro Barba immediately following the campaign. The pie chart inset illustrates the overall sterilization rate achieved, with 81.4% of the community cat population sterilized and 18.6% remaining unsterilized post-intervention.

Globally, the intervention significantly shifted the proportion of sterilized versus unsterilized cats. As illustrated in the *inset* of Figure 5, 81.4% of the cat population was sterilized following the campaign, with only 18.6% remaining unsterilized. This shift highlights the substantial impact of the campaign in altering the reproductive potential of the population in a short period.

The effectiveness of the campaign varied by feeding points, as outlined in Table 2 and represented in Figure 5. Feeding points CS-CT1 and CS-CT3 showed high intervention success, achieving sterilization rates of over 90%. Conversely, CS-CT6 exhibited a lower rate due to challenges in collaboration from the caretaker, likely related to personal issues such as mental health or substance dependencies. Other points like CS-CT2, CS-CT4, and CS-CT5 displayed moderate to high sterilization ratios, indicating successful community engagement and strategic trapping efforts. Notably, some points saw increased participation from cat owners, who brought their pets for sterilization, enhancing the overall campaign impact. In Pedro Barba, the results were more uniform due to the smaller and simpler population structure.

A notable incident during the campaign was the death of one adult female cat in the post-operative period. Despite testing negative for infectious diseases and presenting an appropriate body condition, the exact cause of death could not be determined. The body was preserved for transfer to Gran Canaria for a necropsy, but an electrical issue that left the freezer without power for several days led to the deterioration of the carcass, preventing a conclusive examination.

Despite the high number of interventions achieved within a condensed timeframe, logistical delays in securing permits and preparing the campaign led to shifts in the population structure between the May census and the July campaign. Many kittens recorded in May had matured sufficiently by July to be trapped, resulting in a considerable proportion of sterilized individuals being under one year of age, with many younger than six months. This shift left some adult cats uncaptured. Reports from caretakers indicated several fatalities due to traffic incidents, which were identified as the leading cause of cat mortality on the island, despite its status as a protected natural reserve. Additionally, there were accounts of kittens being adopted, although precise figures were not provided.

**Figure 6:**
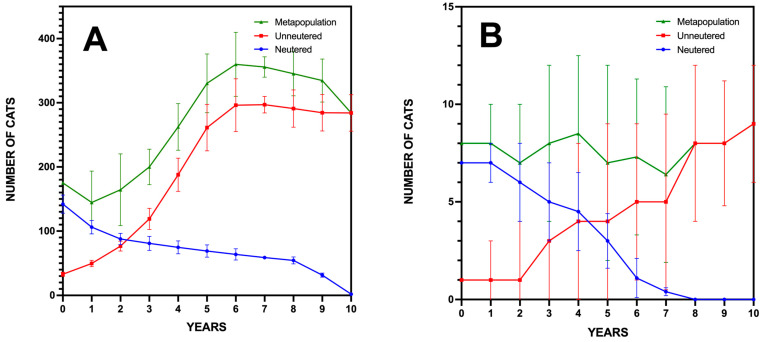
Population Viability Analysis (PVA) projections over 10-year period for community cats’ populations in Caleta de Sebo (**A**) and Pedro Barba (**B**) after sterilization campaign as single action (without further management).

The PVA results for Caleta de Sebo revealed an initial decline in the population size, likely influenced by the inherent high mortality rate of unowned cats. However, as depicted in Figure 6A, the remaining pool of reproductive individuals was sufficient to sustain population growth. Over time, the effect of the mass sterilization began to diminish, and by approximately the second to third year, the number of unsterilized cats surpassed that of the sterilized ones. This shift would lead to an exponential population increase, eventually offsetting the initial impact of the sterilization campaign. The population growth rate (r = 0.343) and its standard deviation (SD(r) = 0.205) reflected this dynamic, with no risk of extinction observed (Pr. Extinction = 0.00). The projected mean population size (N = 279) indicated a return to pre-intervention levels over the 10-year period.

In Pedro Barba, the PVA results demonstrated a more straightforward trajectory. The population growth rate (r = 0.022) was lower, and the standard deviation (SD(r) = 0.004) suggested minimal annual variability. The probability of extinction was notable at 0.32, indicating a significant change within the decade. The mean population size after ten years (N = 10) reflected a pattern of stabilization followed by a gradual decline, as is consistent with the smaller and more isolated nature of this settlement, probably through immigration of cats or the reproduction of the only unneutered female remaining (Figure 6B).

### 3.3. Post-Campaign Population Dynamics

Following the mass sterilization campaign in July 2024, a second maintenance phase was initiated. An experienced volunteer remained on the island to update the census, identify unsterilized cats, and habituate them to human presence using highly palatable food. The aim was to achieve a sterilization rate as close to 100% as possible. In addition to the cats within the main intervention area, we precisely identified the community cats inhabiting peri-urban zones for which permission to trap and sterilize was denied at the outset of the study. This group comprised an additional 19 adult animals: 8 located at the island’s temporary waste disposal site (garbage compactor) and 11 across various agricultural plots dispersed throughout the natural park. These individuals were considered relatively easy to trap due to their known locations and the effectiveness of highly palatable food combined with advanced trapping techniques.

However, the continuation of the project became unfeasible due to unforeseen administrative challenges, leading to the withdrawal of surgical equipment and the decision not to establish the temporary clinic. Despite the halted interventions, the ongoing census from mid-August to November 6 documented the birth of 19 new kittens. It is possible that some of these kittens were already born during the initial intervention period, as lactating females were observed at that time. As of November 6, two additional females were in advanced stages of pregnancy, with an estimated five to seven viable kittens expected in the coming weeks.

Mortality data during this period indicated four deaths within the cat population. One cat died a week post-surgery due to complications; this individual was in poor condition (body condition score of 4) and heavily infested with parasites at the time of sterilization. The remaining three fatalities resulted from traffic incidents, including two young kittens, highlighting traffic as a leading cause of mortality on the island. Six of the newborn kittens were successfully adopted. Two cats were captured by park authorities; one was culled (contrary to legal stipulations) due to its “ferality”, while the other remained confined in a municipal shelter due to administrative complexities.

**Figure 7:**
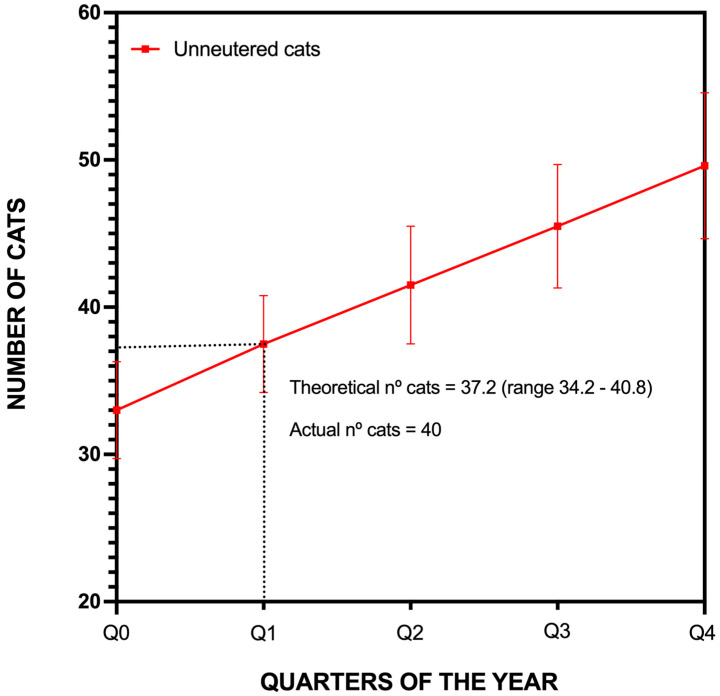
Comparison between observed and projected population growth in La Graciosa during first three months post-intervention based on Population Viability Analysis (PVA) model.

By the conclusion of the study, there was a net increase of seven cats over the three-month period. With the impending births from the two pregnant females, an additional five to seven kittens were anticipated, potentially further increasing the population. To contextualize this short-term increase, we compared the observed growth rate with the projections of the Population Viability Analysis (PVA) model. Figure 7 illustrates how the actual population increase aligns with modeled expectations over the same period, providing a direct comparison of real and predicted population trends.

**Figure 8:**
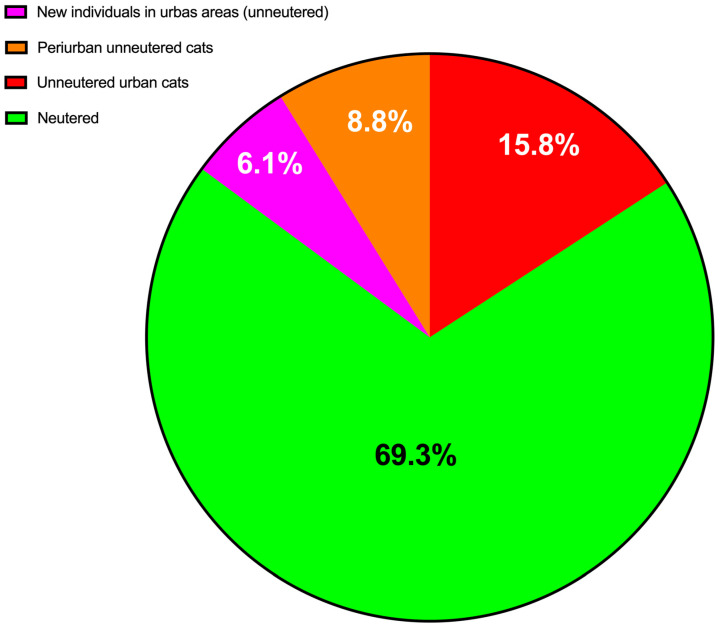
Real proportion of sterilized and unsterilized cats in different categories of Island of La Graciosa following intervention.

The 19 unsterilized adult cats in the peri-urban areas, identified but not yet intervened upon due to lack of permissions, represent a significant reservoir for future population growth. Consequently, the overall sterilization rate decreased to 69.3% (Figure 8). Their known locations and the feasibility of trapping them underscore the importance of addressing these groups in any comprehensive population management strategy.

Finally, we performed a theoretical additional PVA, assuming that the second phase was completed and had yielded the targeted 93% sterilization data (initial population = 222 cats; 207 unowned and 15 owned with outdoor access, according to our updated census) and that annual maintenance is performed by the municipality. In the model, we assumed that this maintenance consists of six newborn cats from the pool of unneutered cats are either sterilized or given in adoption annually (from years 1 to 4), and that seven cats (either sterilized or not) were trapped and relocated out of La Graciosa due to proven high predation rates of these individuals as permitted by law 7/2023. Since La Graciosa’s cat population is isolated, as in previous PVA models, we assume no immigration and no abandonment of new cats.

**Figure 9:**
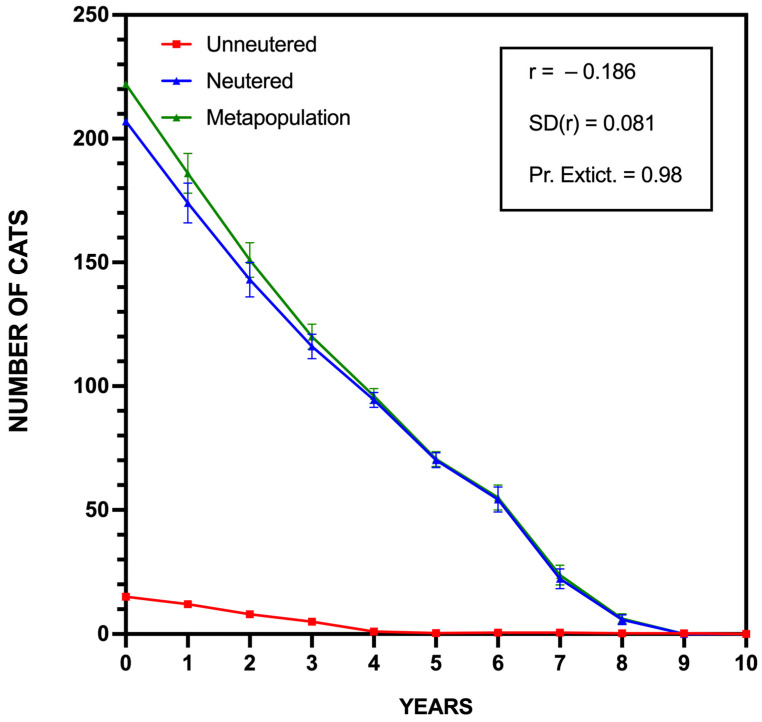
Hypothetical Population Viability Analysis (PVA) for La Graciosa’s community cats under sustained management with 93% sterilization rate and theoretical annual maintenance.

The results of this model are presented in Figure 9, which gives a probability of extinction of community cats of 98% in La Graciosa in a 10-years period, with the disappearing of the unneutered cats subpopulation at year 4, and of neutered community cats at year 8.5.

## 4. Discussion

The initial outcomes from the July 2024 TNR campaign on La Graciosa underscore a substantial reduction in the reproductive potential of the island’s community cat population, as evidenced by an 81.4% sterilization rate achieved in the urban settlements within just three days. This success was largely due to the thorough prior census, conducted with the active involvement of the local community, which played a crucial role in locating cats and assisting with their capture. Such a high sterilization coverage is directly linked to an immediate drop in potential births, particularly through the prevention of new litters and the reduction in gestation and lactation cases, which are significant contributors to rapid population growth. While behavioral changes related to predation may take longer to manifest, this reduction in reproductive activity contributes to a progressive modulation of predation dynamics, gradually reducing the pressure on native biodiversity over time [38,40,41]. By reducing reproductive rates, TNR interventions like this can stabilize population dynamics in ecologically sensitive areas, which become particularly critical in protected zones where non-native predators pose significant threats to vulnerable endemic species [2,3,11,42]. This outcome mirrors findings from previous TNR studies that emphasize the importance of achieving high sterilization rates for effective population control in the short-term [43].

However, while the short-term impacts of TNR are promising, the limitations of this approach become evident without sustained efforts. The PVA projections underscore that ongoing intervention with geographical consistency is essential for sustainable population control in the long-term [43]. According to our PVA, without regular follow-up efforts, the community cat population is likely to rebound within two to three years, or even sooner when considering all cats located throughout the island. This reality underscores the urgency of addressing the historical lack of coordinated efforts to manage the community cats’ population on La Graciosa effectively. For over two decades, despite legal frameworks designed to protect the island’s biodiversity, no substantial measures were implemented, allowing the problem to persist and worsen. It is essential to stress that the ultimate goal of the initiative presented in this paper is not to maintain the presence of community cats on La Graciosa, but rather to implement a pragmatic containment strategy that mitigates ecological damage while more sustainable, long-term solutions are explored. This aligns with the overarching conservation goal of eventually achieving the extinction of community cat populations in this highly sensitive environment. This finding aligns with research by Boone et al. [38] and Benka et al. [40], which also highlights the diminishing efficacy of TNR in the absence of continued sterilization efforts. This makes clear that, while TNR can be an effective tool for immediate population management, isolated interventions are not sufficient for lasting control, particularly in closed ecosystems like La Graciosa where migration and external population inputs are minimal.

While the TNR campaign was a critical first step, its continuity faced significant challenges due to media coverage that amplified concerns raised by local ecologist organizations. These groups criticized the campaign for potentially perpetuating the ecological risks posed by community cats within the Natura 2000 site, with their primary argument centered on the ongoing predation of protected species. Ironically, such objections surfaced after several decades of inaction, during which no meaningful efforts were undertaken to address the growing community cat population or its ecological impacts. Instead of supporting this pragmatic and non-lethal intervention aimed at curbing the problem’s escalation, these groups resorted to public criticism and even threats of legal action, undermining a measure that sought to prevent further population growth and mitigate damage to local biodiversity. This opposition, combined with heightened public scrutiny, led to administrative decisions that ultimately halted the project and prevented the completion of the planned sterilizations, thereby highlighting the deep-seated sociopolitical and institutional barriers that have long hindered biodiversity conservation efforts on the island.

In other contexts, TNR has demonstrated success as a management strategy, particularly in urban areas where community support and municipal resources facilitate sustained efforts [44,45,46]. However, achieving similar outcomes in rural or semi-natural environments presents challenges due to lower human population density, fewer caretakers, and limited resources for sustained follow-up [47]. This is exemplified by La Graciosa, where despite the high sterilization rate achieved, the isolation and limited resources mean that consistent intervention is vital to maintain the initial gains.

La Graciosa represents a complex test case for TNR in ecologically sensitive settings. Unlike urban areas, where TNR has been widely implemented with strong public support, La Graciosa’s protected status as a Natura 2000 site introduces strict conservation priorities that must be reconciled with any management intervention involving non-native predators. This duality presents a unique dilemma: while TNR aligns with non-lethal, welfare-conscious management approaches and complies with Law 7/2023 [28], the conservation mandate to protect endemic species necessitates a delicate balance between humane cat population control and ecosystem preservation. We explicitly recognize that the continued presence of community cats on La Graciosa is incompatible with the island’s ecological sensitivity. The current TNR initiative should therefore be understood as a temporary measure aimed at reducing immediate ecological harm while addressing the long-standing inaction that has characterized conservation efforts in this region. The challenges faced in La Graciosa parallel those observed in other biodiversity-rich islands, where community attitudes play a pivotal role in shaping management strategies.

**Figure 10:**
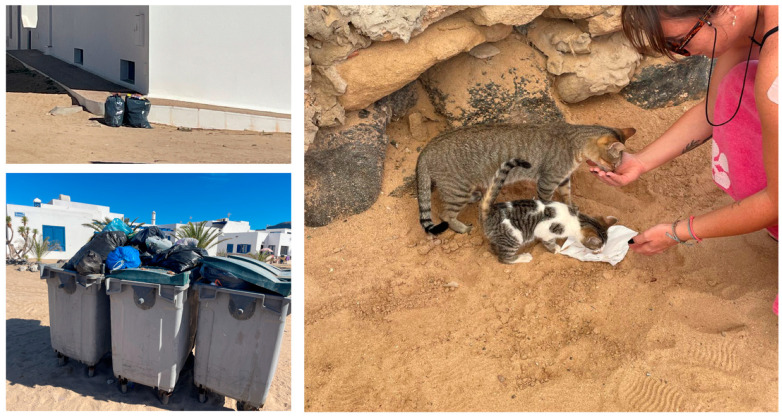
Multiple anthropogenic food sources available to free-roaming cats on La Graciosa. (**Upper left panel**) unsecured garbage bags left in urban areas, which are frequently accessed by cats; (**lower left panel**) open dumpsters filled with organic waste, providing another readily available food source; (**right panel**) direct feeding by tourists, common practice, that reinforces human–cat interactions and complicates enforcement of feeding bans.

For instance, Mameno et al. [48] documented public attitudes toward cat management on Amami Oshima Island, Japan, where there was high acceptance of TNR and adoption but significant resistance to lethal control—particularly among cat owners due to cultural and social values. Similarly, the case of La Palma, also in the Canary Islands, highlights how residents’ perceptions of cats as both pest controllers and companions add another layer of complexity to management efforts [16]. Additionally, a systematic review of cat management practices in remote Indigenous communities in Australia suggests that culturally appropriate, multi-method approaches that combine TNR with education and community involvement yield the most sustainable results, especially in areas with strong social ties to community cats’ populations [49]. On La Graciosa, for example, prohibiting feeding practices was deemed counterproductive, as it could drive cats to expand their ranges into adjacent natural areas in search of prey, exacerbating the ecological impact. Instead, we propose managed feeding with balanced diets rich in animal protein, which research has shown to reduce predation rates and align with conservation priorities, as proposed by other authors [50]. A key factor influencing cat population dynamics in La Graciosa is the abundance of anthropogenic food sources, which range from unsecured garbage bags to direct feeding by tourists and caretakers (Figure 10). This availability of food complicates management strategies, as it sustains the population regardless of formal feeding bans, reinforcing the argument that prohibiting feeding alone would not be an effective measure for population control or mitigating ecological impact.

Research on enforcement-based management practices, such as culling, reveals the substantial psychological impact on caregivers who have formed bonds with community cats. In Newcastle, Australia, lethal cat control at a port led to significant distress among caregivers, some of whom experienced symptoms of traumatic stress [51]. Humane management strategies like TNR not only reduce harm to animals but also alleviate caregiver stress by decreasing anxiety around the potential loss of cats under their care [18,19]. Zito et al. [19] further examine “semi-ownership”, showing that many individuals feel a responsibility toward community cats, even if they do not see them as pets. This underscores the need for humane, non-lethal management approaches like TNR, which align with community sentiments and welfare standards. This underscores the importance of balancing immediate conservation actions with ethical considerations to build public trust and community support for long-term goals, such as the eventual reduction and removal of community cats from sensitive ecosystems [18].

The local community’s strong opposition to the removal of cats reflects a broader shift in societal attitudes within Spain and across Europe, where sensitivity to animal welfare has increased markedly in recent years [16,52,53]. On La Graciosa, however, this sentiment is compounded by the cats’ practical role within the island’s ecosystem and community. Beyond being companion animals, cats are seen as essential in controlling pest populations, a role that residents regard as vital for maintaining ecological balance. This unique intersection of cultural values, animal welfare, and ecological functionality adds further complexity to TNR efforts in such settings, where residents advocate for ethical management practices that align with conservation and community needs.

However, while TNR can serve as an effective initial control measure, it may not suffice for rapid population reduction in biodiversity-sensitive areas. Complementary strategies, such as fostering adoption programs for socialized cats and particularly for kittens within the socialization age, could find support within the community [51]. Additionally, relocating highly predatory cats to designated sanctuaries could be a viable option if such facilities were established. Nevertheless, as of now, there are no suitable sanctuaries or structured relocation plans in place, particularly due to administrative and logistical barriers. This lack of infrastructure underscores the need for practical and ethical solutions that consider the constraints imposed by local resistance and conservation priorities. Further refinement of decision-making could leverage individual genotyping and molecular scatology techniques to assess diet and identify specific cats impacting native species, thus optimizing sanctuary use and conservation outcomes [54,55].

However, translating this nuanced approach into practice has proven challenging. The TNR initiative on La Graciosa not only encountered significant administrative and legal barriers, but it also exposed the coexistence of radically different interpretations of the existing legislative framework. While other islands have successfully implemented full cat removal, these cases typically relied on lethal methods, such as culling, or extensive relocation programs—neither of which are viable under Spain’s legal framework. Law 7/2023 explicitly prohibits lethal management and indefinite confinement, significantly limiting the scope of permissible actions. But, while Law 7/2023 grants cats a protected status as companion animals regardless of their habitat or degree of socialization with humans [28,32], many conservation advocates argue that community cats should be classified as invasive alien species when identified in natural areas, citing other existing legislation that prioritizes the removal of non-native species from protected ecosystems [25,26]. Some authors continue to demand, albeit unrealistically, the culling or permanent confinement of all cats within natural spaces [56,57]. The practicalities of such measures, even if legally feasible, are further complicated by the lack of relocation infrastructure, public resistance to lethal methods, and the logistical challenges of operating in remote natural environments. These conflicting interpretations reflect a deep-seated tension within the policy framework, hindering cohesive management efforts. In this study, the suspension of sterilization efforts following public complaints from ecologist organizations and subsequent media scrutiny underscores the sociopolitical complexities associated with wildlife management in protected areas.

The regulatory deadlock in La Graciosa exemplifies the need for a harmonized policy approach and inter-agency cooperation [58]. The recent directive from the Canary Islands’ government, which mandates that all cats be relocated from protected areas irrespective of their sterilization status, exemplifies a top–down strategy that often disregards the ethical and logistical challenges of implementation. Additionally, the absence of adequate relocation infrastructure and the constraints imposed by Law 7/2023 on indefinite confinement [28,32] highlight the gap between conservation ideals and practical solutions. Achieving “cat-free” natural environments in such a context requires the development of innovative, humane, and logistically feasible management frameworks that align with both legal mandates and conservation priorities.

Our study’s findings underscore the importance of context-sensitive approaches that integrate both ecological and social considerations, including stakeholder engagement, to help align conservation and social considerations.

## 5. Conclusions

This study provides a comprehensive, evidence-based assessment of TNR (Trap–Neuter–Return) as a humane management strategy for community cats within a Natura 2000 protected area. The high sterilization rate achieved in La Graciosa underscores TNR’s rapid impact in reducing reproductive potential, which contributes to alleviating ecological pressures over time, as sterilization gradually reduces reproductive-driven impacts. However, our Population Viability Analysis (PVA) indicates that without sustained, periodic interventions, the cat population is likely to rebound, revealing TNR’s limitations as a standalone solution for long-term population control. As emphasized throughout the manuscript, the ultimate goal of management efforts in La Graciosa is not to perpetuate the presence of community cats, but rather to implement a pragmatic approach that mitigates their ecological impact while exploring long-term solutions in alignment with conservation priorities.

Our findings emphasize the need for a holistic management approach that integrates TNR with additional strategies, such as adoption programs for sociable cats and selective relocation of individuals with high predation rates. Advanced genetic tools, like individual genotyping and molecular scatology, offer promising avenues to refine these strategies. By allowing the identification of specific predatory individuals, these techniques would support data-driven decision-making for relocation efforts, thereby enhancing TNR’s efficacy and ecological alignment. Moreover, community engagement and education remain critical components of successful implementation, ensuring public support for humane and science-based solutions.

The case of La Graciosa also illustrates the complex intersection between conservation goals and ethical management practices, especially in regions where local communities oppose lethal control measures. This study highlights the importance of addressing not only the ecological impacts of community cats but also the social and cultural dimensions of their management, demonstrating that integrated, multifaceted approaches are more likely to succeed in balancing conservation and welfare priorities. It also highlights the urgent need to address historical inaction, which has allowed community cat populations to persist in sensitive ecosystems despite existing biodiversity protection laws. This study represents the first significant attempt to reverse this trend in La Graciosa, laying the groundwork for future integrated strategies.

In Spain, the coexistence of Law 7/2023, which endorses humane cat management, with conservation laws that prioritize native species protection, reflects a regulatory conflict that demands harmonization. This regulatory gap underscores the need for inter-agency cooperation and long-term planning to ensure that conservation efforts are not undermined by conflicting interpretations of the law. Addressing these discrepancies through inter-agency cooperation will be crucial to ensuring that conservation strategies are both effective and legally viable. This study highlights the potential and limitations of TNR within this context, suggesting that effective population control in ecologically sensitive areas requires a multifaceted strategy that combines TNR with additional management measures, ensuring both biodiversity conservation and animal welfare. Overcoming administrative and regulatory challenges will be essential, as these obstacles significantly impact intervention efficacy in protected areas. Ultimately, this study underscores the critical importance of integrating ecological, scientific, and ethical considerations to address the challenges of managing non-native predators in biodiversity-sensitive areas. The insights gained from La Graciosa can inform broader conservation frameworks, ensuring that both biodiversity and animal welfare priorities are met in a balanced and sustainable manner.

## Figures and Tables

**Table 1:** The cats recorded in the initial census in the urban nucleus of La Graciosa.

**Caleta de Sebo**				
**Community Cats**				
Code *	**N° Cats in the Census**	**Unneutered**	**Neutered**	**% Neutered**
CS-CT1	18	7	11	61.1
CS-CT2	17	13	4	23.5
CS-CT3	16	13	3	18.8
CS-CT4	17	17	0	0.0
CS-CT5	3	3	0	0.0
CS-CT6	19	16	3	15.8
CS-CT7	7	1	6	85.7
CS-CT8	8	7	1	12.5
CS-NCT1	25	25	0	0.0
CS-NCT2	22	22	0	0.0
**Totals**	**152**	**124**	**28**	**18.4**
**Owned Cats**				
Code *	**N° Cats in the Census**	**Unneutered**	**Neutered**	**% Neutered**
CS-O1	1	0	1	100.0
CS-O2	1	1	0	0.0
CS-O3	3	0	3	100.0
CS-O4	4	2	2	50.0
CS-O5	1	0	1	100.0
CS-O6	4	4	0	0.0
CS-O7	1	1	0	0.0
CS-O8	1	1	0	0.0
CS-O9	2	1	1	50.0
CS-O10	1	1	0	0.0
CS-O11	1	1	0	0.0
CS-O12	1	1	0	0.0
CS-O13	1	0	1	100.0
CS-O14	1	1	0	0.0
CS-O15	1	1	0	0.0
**Totals**	**24**	**15**	**9**	**62.5**
**Pedro Barba**				
Code *	**N° Cats in the Census**	**Unneutered**	**Neutered**	**% Neutered**
PB-CT1	8	8	0	0.0

* CS-CT: Caleta de Sebo, feeding point with caretaker; CS-NCT: Caleta de Sebo, feeding point without caretaker; CS-O: Caleta de Sebo, cats with a self-declared owner; PB-CT1: Pedro Barba, feeding point with caretaker.

**Table 2:** Sterilization ratios per feeding points following mass sterilization campaign in urban areas of La Graciosa.

**Caleta de Sebo**				
**Community Cats**				
Code *	**N° Cats in the Census**	**Unneutered**	**Neutered**	**% Neutered**
CS-CT1	18	2	16	88.9
CS-CT2	17	2	15	88.2
CS-CT3	16 (−1) **	4	12 (−1) **	75.0
CS-CT4	17	2	15	88.2
CS-CT5	3	0	3	100.0
CS-CT6	19	8	11	57.9
CS-CT7	7	0	7	100.0
CS-CT8	8	0	7	87.5
CS-NCT1	25	6	19	76.0
CS-NCT2	22	7	15	68.2
**Totals**	**151**	**31**	**119**	**78.9**
**Owned Cats**				
Code *	**N° Cats in the Census**	**Unneutered**	**Neutered**	**% Neutered**
CS-O1	1	0	1	100.0
CS-O2	1	0	1	100.0
CS-O3	3	0	3	100.0
CS-O4	4	0	4	100.0
CS-O5	1	0	1	100.0
CS-O6	4	0	4	100.0
CS-O7	1	0	1	100.0
CS-O8	1	0	1	100.0
CS-O9	2	1	1	50.0
CS-O10	1	0	1	100.0
CS-O11	1	1	0	0.0
CS-O12	1	0	1	100.0
CS-O13	1	0	1	100.0
CS-O14	1	0	1	100.0
CS-O15	1	0	1	100.0
**Totals**	**24**	**2**	**22**	**91.6**
**Pedro Barba**				
Code *	**N° Cats in the Census**	**Unneutered**	**Neutered**	**% Neutered**
PB-CT1	8	1	7	87.5

* CS-CT: Caleta de Sebo, feeding point with caretaker; CS-NCT: Caleta de Sebo, feeding point without caretaker; CS-O: Caleta de Sebo, cats with self-declared owner; PB-CT1: Pedro Barba, feeding point with caretaker. ** One female died suddenly in post-operatory room.

## Data Availability

The data supporting the reported results are available upon request from the corresponding author. Due to ethical and privacy considerations, the data are not publicly accessible.

## Supplementary Materials

The following supporting information can be downloaded at: https://www.mdpi.com/article/10.3390/ani15030429/s1, Table S1: Key Parameters and Assumptions for PVA models in La Graciosa.
