# Correction: Luzardo et al. Integrating Conservation and Community Engagement in Free-Roaming Cat Management: A Case Study from a Natura 2000 Protected Area. *Animals* 2025, *15*, 429

**DOI:** 10.3390/ani15121683

**Published:** 2025-06-06

**Authors:** Octavio P. Luzardo, Andrea Hansen, Beatriz Martín-Cruz, Ana Macías-Montes, María del Mar Travieso-Aja

**Affiliations:** 1 Research Institute of Biomedical and Health Sciences (IUIBS), University of Las Palmas de Gran Canaria, Paseo Blas Cabrera “Físico” s/n, 35016 Las Palmas de Gran Canaria, Spain; beatriz.martin@ulpgc.es (B.M.-C.); ana.macias@ulpgc.es (A.M.-M.); marimar.travieso@ulpgc.es (M.d.M.T.-A.); 2 Spanish Biomedical Research Center in Physiopathology of Obesity and Nutrition (CIBERObn), 28029 Madrid, Spain; 3 Association for Respect and Commitment to Animals and Nature (ARYCAN), 35217 Valsequillo de Gran Canaria, Spain; arycan.ah@gmail.com

## Error in Figure

In the original publication [1], there was a mistake in Figure 5 as published. Due to the authors’ oversight during the proofreading stage, the published figure shows an incorrect image and does not correspond with the figure legend. Figure 5 of the published paper should be replaced with the correct figure showing the effectiveness of the July 2024 sterilization campaign in La Graciosa, Canary Islands.

The corrected Figure 5 appears below. The authors state that the scientific conclusions are unaffected. This correction was approved by the Academic Editor. The original publication has also been updated.

## Figures and Tables

**Figure 5:**
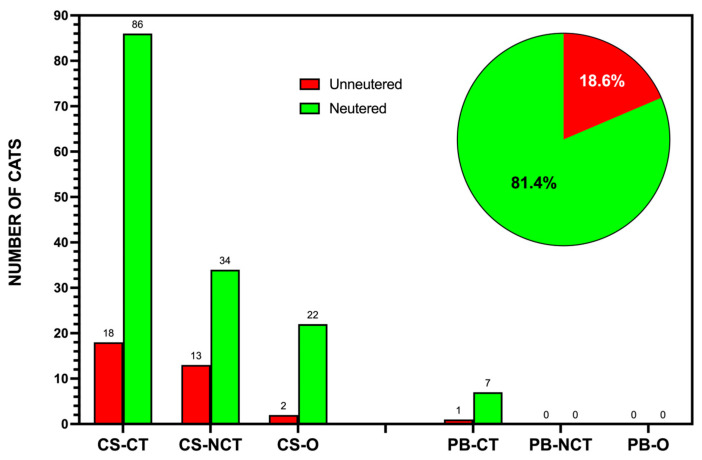
The effectiveness of the July 2024 sterilization campaign in La Graciosa, Canary Islands. The bar chart shows the number of neutered (green) versus unneutered (red) cats recorded across different feeding points in Caleta de Sebo and Pedro Barba immediately following the campaign. The pie chart inset illustrates the overall sterilization rate achieved, with 81.4% of the community cat population sterilized and 18.6% remaining unsterilized post-intervention.
