# Supplementary material for: Integrating Conservation and Community Engagement in Free-Roaming Cat Management: A Case Study from a Natura 2000 Protected Area
Source: Animals (Basel). 2025 Feb 4;15(3):429. doi: 10.3390/ani15030429 (PMC11815770; doi:10.3390/ani15030429)
Supplement: Supplementary file 1 [file animals-15-00429-s001.zip › animals-3448952-supplementary.pdf]

animals

MDPI

# Supplementary Materials:

**Supplementary Table S1: Key Parameters and Assumptions for PVA models in La Graciosa**

| Parameter | Value/Assumption | Justification |
|---|---|---|
| **Carrying Capacity** | 460 cats | Fixed to reflect the worst-case scenario as 2.5 times the initial census of cats in La Graciosa. |
| **Reproductive Rates** | 3 litters/year per breeding female | Reflects continuous reproduction due to the island's warm climate (no seasonal interruption). |
| **Litter Size** | 3.5 kittens per litter (average) | Empirical data from field observations and literature for urban cat populations. |
| **First-Year Mortality Rate** | 35% (both sexes) | High mortality rate due to environmental stressors such as predation, disease, and starvation. |
| **Reproductive period (females)** | 6 months to 8 years | Represents the age range during which females are reproductively active in most cases |
| **Reproductive period (females)** | 5 months to 9 years | Represents the age range during which females are reproductively active in most cases |
| **Mortality Rate (Subsequent)** | 10% (females), 15% (males) per year | Reflects higher male mortality due to territorial disputes and roaming behaviors. |
| **Maximum lifespan** | 10 years | Based on observations from the TNR programs worlwide, most cats die before reaching 6 years old, but in La Graciosa, the level of threaths faced by the cats is lower, so higher lifespan can be predicted |
| **Sterilization Impact** | 100% reduction in reproduction for sterilized individuals | Modeled as a catastrophic event, completely halting reproduction in sterilized subpopulations. |
| **Disease Outbreaks** | Viral incidence: 5% annually; mortality: >50% | Modeled as stochastic catastrophic events to simulate potential epidemics (e.g., calicivirus, panleukopenia). |
| **Immigration and Abandonment** | 0% annually | Due to the isolation of the island this paramete,, which is very important in other settings, can be ignored in this scenario. |
| **Adoption rate** | 1.5% annually | Included as a 'Harvest' parameter reflecting adoption of some kitten as a minor but consistent population decrease factor. |
| **Trap and removal for culling or confinement** | 1% annually | This is based on reported records from the last years, and some registered, even after the approval of Law 7/2023, which explicitly prohibits such practices |
| **Euthanasia** | <1% annually. Based on veterinary records | Includes humane euthanasia recorded during monitoring. Modeled under 'Harvest' |
